# Preoperative workup in the assessment of adrenal incidentalomas: outcome from 282 consecutive laparoscopic adrenalectomies

**DOI:** 10.1186/1471-2482-13-57

**Published:** 2013-11-27

**Authors:** Mario Musella, Giovanni Conzo, Marco Milone, Francesco Corcione, Giulio Belli, Maurizio De Palma, Annunziato Tricarico, Luigi Santini, Antonietta Palazzo, Paolo Bianco, Bernadette Biondi, Rosario Pivonello, Annamaria Colao

**Affiliations:** 1 General and Emergency Surgery, “Federico II” University, Naples, Italy; 2 General and Endocrine Surgery, Second University “SUN”, Naples, Italy; 3 General and Laparoscopic Surgery, AORN “Monaldi” Hospital, Naples, Italy; 4 General Surgery, “S. Maria di Loreto Nuovo” Hospital, Naples, Italy; 5 General and Endocrine Surgery, AORN “A. Cardarelli” Hospital, Naples, Italy; 6 General and Emergency Surgery, AORN “A. Cardarelli” Hospital, Naples, Italy; 7 Endocrinology, “Federico II” University, Naples, Italy

**Keywords:** Laparoscopic adrenalectomy, Incidentaloma, Adrenocortical cancer, Preoperative workup, Pheochromocytoma, Guidelines

## Abstract

**Background:**

To confirm the efficacy of preoperative workup, the authors analyse the results of a multicentre study in a surgical series of patients diagnosed with an adrenal incidentaloma.

**Methods:**

The retrospective review of a prospectively collected database was conducted. The data was obtained by six surgical units operating in the Campania Region, Italy. Five-hundred and six (506) adrenalectomies performed between 1993 and 2011 on 498 patients were analysed. Final histology in patients with a preoperative diagnosis of incidentaloma and studied according to guidelines (230/282 patients group A) was compared with final histology coming from patients presenting the same preoperative diagnosis but studied not according to guidelines (52/282 patients group B).

**Results:**

In group A preoperative diagnosis was confirmed at final histology in 76/81 (93.8%) cases of subclinical functioning lesions presenting as an incidentaloma. The preoperative detection of pheochromocytoma and primary adrenocortical cancer (ACC) reached 91.6% and 84.6% respectively. In group B conversion rate to open surgery was higher than in group A (p = 0.02). One pheochromocytoma was missed at preoperative diagnosis whereas one ACC smaller than 4 centimetres (cm) and coming from an incidental lesion was discovered. In both groups a significant association between increasing dimensions of incidentaloma and cancer has been observed (p = 0.001).

**Conclusions:**

This surgical series confirm the high efficacy of suggested guidelines. A significant preoperative detection rate of adrenal lesions presenting as incidentaloma is observed. The unnecessary number of adrenalectomies performed in understudied patients, causing higher morbidity, was not associated to a higher detection rate of primary adrenocortical cancer.

## Background

The term “incidentaloma” is widely used to define the age increasing prevalence of incidentally discovered adrenal masses in 3 to 10% of patients undergoing imaging modalities, mainly CT-scan and magnetic resonance imaging (MRI), for other clinical indications [1,2]. These neoplasms represent the most frequent adrenal tumours encountered by physicians and they are accompanied with a negative clinical functional status, thus they are often defined as “challenging” in terms of preoperative diagnosis and management [1]. This is due to both their imaging characteristics, that sometimes do not permit the radiologists to formulate a diagnosis, and because fine needle aspiration biopsy (FNAB) does not always allow to distinguish benign from malignant primary adrenocortical tumours [3].

Despite these challenges, the goal of the clinicians dealing with an adrenal incidentaloma remains to remove functioning and malignant lesions without performing an useless adrenalectomy in non-functioning small benign tumours. In support of this approach, the most exhaustive guidelines concerning the correct management of adrenal incidentalomas have been proposed by the United States National Institute of Health (NIH) and by the American Association of Clinical Endocrinologists/American Association of Endocrine Surgeons (AACE/AAES) [4,5]. When an adrenal incidentaloma is detected, according to these statements, the main clinical aims are to rule out the occurrence of any subclinical cortisol or aldosterone secreting lesion, to exclude a pheochromocytoma, and, finally, to resect all adrenal non-functioning lesions suspicious for adrenocortical cancer (ACC) at imaging.

If a secreting lesion is detected, the indications to treatment vary from a more aggressive attitude for pheochromocytoma to a non surgical management to be preferred in some cases of cortisol or aldosterone subclinical secreting lesions [5]. Additionally, two more points are clearly established. Firstly, adrenal masses identified as potential metastases during the staging of an extra adrenal cancer should be not considered as incidentalomas; secondly, most non secreting lesions larger than 4 cm must be removed due to the increased risk of an ACC irrespectively of their imaging status [5].

In this last decade laparoscopic adrenalectomy has gained gold standard status. It is widely used to resect almost all adrenal lesions, limiting open surgery to specific indication [6]. Although several experts have tried to define the ideal management of incidentalomas reaching the conclusion that, regardless of the surgical approach, open or laparoscopic, the indications to surgery through the years have not changed [7-10], some articles have demonstrated that the introduction of laparoscopy has caused an increase in the number of adrenalectomies performed on patients with incidentalomas [11-13]. In our study the final histology of 282 consecutive laparoscopic adrenalectomies performed at different institutions for incidentaloma, was evaluated. Patients have been then divided in two groups. Group A included patients who received a preoperative workup according to protocols [4,5], while patients whose pre-surgical workup did not follow current guidelines were included in group B.

The aims of this study were:

1. To define in both groups in which rate preoperative work-up has matched final diagnosis when an incidentaloma was detected, especially for suspected pheochromocytoma and primary ACC.

2. To determine if unnecessary adrenalectomies in group B led to a higher rate of discovery of incidental cancer, thus suggesting a possible shift in surgical indications.

3. To compare if morbidity in patients who received a preoperative workup according to protocols (Group A) was significantly different from that of understudied patients (Group B).

## Methods

### Study setting and timing

To investigate the three points defined in the introduction, a questionnaire was prepared by four authors (MM, BB, RP, AC) working at the “Federico II” University and e-mailed at the end of 2011 to surgical units with an experience of more than fifty laparoscopic adrenalectomy operating in the Campania Region, Italy. Seven surgeons from six centres participated in this audit: 2 university centres (1 from Naples Second University “SUN” and 1 from Naples “Federico II” University), and 3 regional hospitals with four centres. The duration of data analysis ranged from January 1993 to December 2011. During this period 506 laparoscopic adrenalectomies (LA) were performed on 498 patients. Male patients were 177 (35.5%). Mean age was 49.7 (range 16–78). In 282 cases, (55.7%) the resection was performed due to a preoperative diagnosis of adrenal incidentaloma. All six centres began, starting from 2001, a strict collaboration with the endocrinology unit of the “Federico II” University in terms of biochemical assessment, imaging evaluation and indication to surgery of detected incidental lesions.

### Study design

The retrospective review of a prospectively collected database was conducted. The main parameters asked in the questionnaire defined:

1. The preoperative functional status and the dimensions of the lesions obtained by biochemical study, CT scan with or without contrast, MRI, radionuclide scanning (MIBG, FDG PET, PET-CT or NP-59) or eventually FNAB.

2. Indications to surgery

3. The surgical records in terms of operative time, number of ports used and intraoperative complications, eventually requiring conversion to open surgery.

4. The postoperative records in terms of duration of hospital stay, complications eventually requiring re-intervention, final dimension and histology of the specimen, and survival data for malignant lesions.

All data recorded later than 1999 were saved on electronic sheets under the supervision of the surgical units heads. Part of the data was collected manually before that period. All records, once returned, were then introduced in a database under the supervision of the authors of the questionnaire for computer processing. A data quality control was performed at that time.

According to study aim, patients were then classified and divided in two groups.

When preoperative diagnosis was “incidentaloma” (282/506 adrenalectomies), only 230/282 lesions (81.5%) underwent, at the time of diagnosis, both a biochemical and radiological investigation, performed by the same endocrinologists group, acting as coordinator. The study, according to guidelines [5], was aimed to:

Rule out subclinical cortisol secreting lesion by an overnight 1 mg dexamethasone suppression test. A response providing values lower than 5 μg/dL was considered normal.

Rule out any subclinical aldosterone secreting lesion by evaluating serum potassium and the plasma aldosterone concentration/plasma renin activity ratio. The cut-off was considered a plasma aldosterone concentration-plasma renin activity ratio greater than 30 and a plasma aldosterone concentration greater than 0.5 nmol/L (20 ng/dL).

Evaluate 24-hour total urinary metanephrines and fractionated catecholamines (or both plasma and urine study) to exclude a pheochromocytoma. A 24-hour urine total metanephrine level above 1,800 μg and a plasma metanephrine level exceeding 3 times normal were assumed diagnostic for a pheochromocytoma.

Conversely, once defined lesions smaller than 4 cm, with benign non contrast CT scan features, all non-functioning lesions looking indeterminate or malignant, were evaluated by using a contrast enhanced CT scan or alternatively in 38/230 cases by MRI. In 25/230 cases a PET-CT scan was however used due to the inconclusive response of CT scan [14]. An imaging pattern not fulfilling the CT scan criteria although suggestive of malignancy was defined indeterminate. The criteria used, indicating a primary ACC in non-functioning incidentalomas were [9,15,16]:

• An attenuation value higher than 10 HU on non contrast CT scan

• A contrast agent washout lesser than 50%

• Dimension bigger than 6 cm.

• Irregular shape, central necrotic areas, vena cava thrombosis.

Indication to surgery in this group of incidentally discovered lesions were then considered:

• Non functioning lesions larger than 4 cm (76 patients)

• Non functioning lesions smaller than 4 cm but increasing in radiographic dimensions or becoming hormonally active within the first year from diagnosis (75 patients)

• Monolateral aldosterone producing masses (18 patients)

• Hormonally active pheochromocytomas (22 patients)

• Patients younger than 40 presenting with a subclinical Cushing syndrome [17] of recent onset and accompanied by worsening of hypertension, abnormal glucose tolerance and osteoporosis (39 patients)

These patients studied and treated according to guidelines, were classified and included in group A.

The remaining 52/282 (18.5%) lesions were preoperatively studied elsewhere with incomplete imaging and underwent surgical resection without further biochemical testing. Most of them (49/52, 94.2%) were treated from 1993 to 2000, at the beginning of the laparoscopic adrenalectomy experience. Incomplete imaging means CT scan performed without contrast when requested or without measuring contrast washout kinetics [4,5]. In these patients, classified as group B, MRI or nuclear scanning were never used. The lack of any clinical manifestation of endocrine activity in presence of an incidental adrenal lesion, evaluated as resectable at imaging, was considered a valid indication to surgery. They, according to the authors of the questionnaire, did not fulfill the preoperative study criteria requested to be included in group A.

All incidental lesions presenting an elevation of 24-hours urinary metanephrines and fractionated catecholamines in group A were treated with an α adrenergic blockade before resection. Subtotal adrenalectomies were not performed. Six patients (1.2%) were approached in supine transabdominal position. The posterior retroperitoneal approach was not used. The “Federico II” University ethical committee, which is the reference centre for our region, authorized the use of all data concerning this study. Before surgery, all patients signed an informed consent, requested by Italian laws and granted by our ethical committee, explaining in detail all the risks and the benefits provided by adrenalectomy.

Statistical analysis was performed with SPSS version 14.0 (SPSS^©^, Chicago, IL, USA). The Yates corrected χ^2^ test was used as a means of evaluating differences in category variables, and the independent sample test was used for continuous variables. Significance was assigned at a level of p <0.05.

## Results

No deaths occurred in this audit. The general morbidity was 7.8% (22/282), see Table 1. The mean hospital stay was 4.1 days in group A patients vs. 4.6 days in group B (p = ns). Conversion rate to open surgery was significantly higher in group B (6/52 11.5%) than in group A (8/230 3.47%) (p = 0.02). Table 2 refers to group A (230/282 81.5%) whereas Table 3 reports final histology for group B (52/282 18.5%) incidental lesions. Among the lesions studied according to guidelines [4,5], final histology matched the preoperative diagnosis for all (100%) subclinical cortisol and aldosterone secreting lesions. Remaining in the field of incidental functioning lesions it must be noted that:

• 24/230 (10.8%) biochemically tested lesions in group A and 1/52 (1.9%) untested lesions in group B were pheochromocytomas at final histology (the patient with postoperative uncontrolled change in blood pressure).

• 19/24 pheochromocytomas (79.1%) in group A presented an elevation of 24-hours urinary metanephrines and fractionated catecholamines higher than twice the upper limit. This was a significant association (p = 0.001).

• 3/24 pheochromocytomas (12.5%) in group A presented an elevation of 24-hours urinary metanephrines and fractionated catecholamines which was lower than twice the upper limit. A significant association was also observed (p = 0.001).

• 2/24 pheochromocytomas (8.3%) in group A presented with normal 24-hours urinary metanephrines and fractionated catecholamines levels.

• 1/4 (25%) ganglioneuromas at final histology presented an elevation of 24-hours urinary metanephrines and fractionated catecholamines levels. It was lower than twice the upper limit.

**Table 1:** Morbidity rate in 282 laparoscopic adrenalectomies for incidentaloma with final histology

**Complication**	**Group A**	**Group B**
Intraoperative bleeding	3 ACC > 6 cm *; 1 myelolipoma > 6 cm*	3 nonfunctioning adenomas < 4 cm*; 1 ACC > 4 cm*
Cava vein tear	1 ACC > 6 cm*	
Liver injury	2 ACC > 6 cm*	1 non functioning adenoma < 4 cm*
Spleen injury	1 myelolipoma > 6 cm*	1 non functioning adenoma < 4 cm*
Postoperative bleeding	2 ACC > 6 cm**	2 nonfunctioning adenomas < 4 cm**
Port site hematoma	1 nonfunctioning adenoma > 6 cm	1 myelolipoma
Port site infection	1 myelolipoma > 6 cm	
Postoperative uncontrolled blood pressure		1 pheochromocytoma
Total	12/230 (5.2%)	10/52 (19.2%)

*Conversion to open surgery required.

**In one case surgical revision required.

**Table 2:** Postoperative diagnosis for 282 laparoscopic adrenalectomies performed for incidentaloma with specimen dimensions

	**lesions < 4 cm**	**lesions 4–6 cm**	**lesions > 6 cm**
Non functioning adenoma	63(56.2)	28(35.8)	10(25)
Cortisol secreting adenoma	20(17.8)	15(19.2)	4(10)
Aldosterone secreting adenoma	8(7.1)	7(8.9)	3(7.5)
Pheochromocytoma	11(9.8)	10(12.8)	3(7.5)
Adrenocortical cancer	---------	1(1.2)	10 (25)
Myelolipoma	8(7.1)	13(16.6)	5(12.5)
Ganglioneuroma	2(1.7)	1(1.2)	1(2.5)
Hematoma	---------	---------	1(2.5)
Cyst	---------	3(3.8)	3(7.5)
Total	112(48.6)	78(33.9)	40(17.3)

Group A, 230/282 patients studied according to guidelines.

Dimensions, n. (%) of adrenalectomies.

**Table 3:** Postoperative diagnosis for 282 laparoscopic adrenalectomies performed for incidentaloma with specimen dimensions

	**lesions < 4 cm**	**lesions 4–6 cm**	**lesions > 6 cm**
Non functioning adenoma	25(48)	4(7.6)	14(26.9)
Cortisol secreting adenoma	-	-	-
Aldosterone secreting adenoma	-	-	-
Pheochromocytoma	1(1.9)	-	-
Adrenocortical cancer	1(1.9)	1(1.9)	-
Myelolipoma	2(3.8)	-	1(1.9)
Ganglioneuroma	-	-	-
Hematoma	-	2(3.8)	-
Cyst	1(1.9)	-	-
Total	30(57.6)	7(13.4)	15(28.8)

Group B, 52/282 patients not studied according to guidelines.

Dimensions, n. (%) of adrenalectomies.

However, by considering non-functioning lesions preoperatively classified as incidentaloma it was observed how:

• 8/13 ACC (61.5%) had a malignant imaging pattern showing both an attenuation value higher than 10 Hounsfield units (HU) at noncontrast CT-scan and a late washout at contrast CT-scan. All lesions were larger than 6 cm.

• 3/13 ACC (23%) had an indeterminate diagnosis at imaging. One was a 5.2 cm lesion at CT scan whereas the remaining two were lesions larger than 6 cm.

• 2/13 ACC (15.3%) showed a benign pattern at imaging. One was a 2 cm lesions at CT scan whereas the second one size was 4.5 cm. Both came from the 52/282 understudied lesions (Group B) described in Table 3.

Globally we found 1 stage I (T1, N0, M0), 9 stage II (T2, N0, M0) and 1 stage III (T3, N0, M0) cancers in group A. Conversely, 2 stage I cancers were found in group B.

In general, if we consider all non-functioning incidental lesions (group A + B) with suspicious imaging features (ACC vs. non-functioning benign adenomas) we observe that 1/89 (1.12%) lesion lesser than 4 cm, 2/34 (5.8%) lesions between 4 and 6 cm and 10/36 lesions larger than 6 cm (27.7%) were primary ACC. In general increasing dimensions were significantly associated with ACC (p = 0.001). However, if we compare lesions bigger than 6 cm with lesions between 4 and 6 cm and with lesions smaller than 4 cm, a significant correlation with cancer is shown (p = 0.03 and p = 0.01 respectively), whereas by comparing lesions between 4 and 6 cm with lesions smaller than 4 cm a significant probability of finding a cancer was not observed (p = 0.08).

## Discussion

This retrospective study, involving six independent surgical units, covers a period of nineteen years. For this reason it is important to highlight limits such as the lack of a central laboratory assessment for some patients, the differences in imaging quality due to technical evolution, the distinct scanning equipment that different centres have used and finally the historical absence of guidelines to treat adrenal incidentalomas before 2002. Furthermore it is strictly a surgical series, in which, if final histology is in any case provided, on the other hand no information is given about incidental lesions that were not resected. Nevertheless, evaluating the points raised in the introduction and observing the results, in our opinion some conclusions might be suggested. By considering 230/282 group A incidentalomas, we found 3/24 (12.5%) pheochromocytomas to have a borderline elevation of metanephrine levels and 2/24 lesions (8.3%) showing normal metanephrine levels, being a pheochromocytoma at final histology. Nevertheless it has to be remarked how in these group of lesions, studied according to protocols [4,5], a preoperative diagnosis for 39/39 cortisol secreting lesions, 18/18 aldosterone secreting lesions and 19/24 (79.1%) pheochromocytomas was obtained. Thus, if we consider only subclinical functioning lesions at final histology presenting as an incidentaloma, a correct preoperative diagnosis was reached in 76/81 (93.8%) lesions. This appears especially important for pheochromocytomas. These lesions must not only be resected [1-5] but diagnosed and treated by an α adrenergic blockade before surgery, to avoid intraoperative hemodynamic instability [18,19].

However, if we consider non-functioning incidental lesions, the preoperative detection rate decreases. In fact, although sometimes suggested, a specific imaging, CT scan or MRI, pattern for adrenal incidentalomas, allowing a preoperative diagnosis, has been never defined [10,20]. Only 8/13 (61.5%) ACC were actually preoperatively addressed by imaging in this series. It may therefore be concluded that, despite the accuracy of preoperative evaluation, about 20.9% of incidental pheochromocytomas and 38.5% of primary cancers miss a preoperative diagnosis. An acceptable 22/24 (91.6%) and 11/13 (84.6%) preoperative detection for pheochromocytoma and primary ACC respectively would however be achieved for both functioning and non-functioning lesions if a further 3/24 borderline pheochromocytomas and 3/13 indeterminate cancers at imaging were considered. These results would also be in line with recent papers [19,21,22] in which both the efficacy of preoperative biochemical testing, or CT and MRI imaging, led preoperative diagnosis to match final histology in a rate ranging from 80 to 90% of resected incidental lesions.

An 18.5% rate (52/282 group B) of lesions classified as “incidental” and described in Table 3 has been on the other hand resected without an accurate preoperative study. This occurred in 49/52 patients (94.2%) treated from 1993 to 2000, when most of the centers participating in our audit were starting their laparoscopic adrenalectomy experience. This disputable attitude has however some plausible explications. The first one, as well as the lack of common endocrine coordination for the participating surgery units, was surely represented by the absence of defined guidelines to treat adrenal incidentalomas before 2002 [4,5]. The second reason was the trend to use small incidental lesions to improve the laparoscopic adrenalectomy learning curve. This attitude, justified by the unpredictable risk of cancer in any non functioning lesion, has been however emphasized in other papers [11-13] to explain the increased number of adrenalectomies that conversely, would have been never performed before the laparoscopic age. A concept supported by the observation that if CT scan tends to underestimate the real dimension of adrenal lesions [23], on the other hand the improvement in imaging quality [11] has allowed the identification of smaller and smaller non functioning lesions. Although this aggressive surgical attitude has led to the detection of one 1/30 (3.3%) adrenocortical cancer among all lesions smaller than 4 cm, it must be highlighted the occurrence of unnecessary resection in 25/52 (48.5%) non-functioning adenomas smaller than 4 cm (Table 3); this value is significantly higher than the 63/230 (27.3%) lesion rate observed in group A (p = 0.005). This result, together with an higher intraoperative complication rate, led to a number of conversion to open surgery significantly higher in group B than in group A. In this perspective, both the overall morbidity rate of 10.9% and the mortality rate of 0.3% described for laparoscopic adrenalectomy [24], have to be reminded. Moreover the preoperative diagnosis of 1/52 (1.9%) subclinical pheochromocytoma has been missed.

While the respect of current guidelines is undisputed about the indication to resect incidental non-functioning lesions larger than 4 cm, the observation that an incorrect protocol on 52/282 patients has led to discover a small incidental cancer in 1/30 lesion lesser than 4 cm (3.3%) should cause some concern. Although our data exceeds previous papers reporting to 2% the detection rate of primary cancer coming from incidental non-functioning lesions smaller than 4 cm. [7-9,25,26], one instance of incidental cancer in a 2 cm lesion is probably not enough to suggest a shift in surgical indication, especially if we consider the entire series of 282 incidentalomas. In that case the cancer rate in incidentalomas smaller than 4 cm would be 1/142 (0.7%), in close agreement with the previously published range. Additionally, according to recent papers, resecting incidental adrenal lesions smaller than 4 cm appears to be not only unnecessary but also cost-ineffective [27]. However in this direction, although a wait and see policy is accepted, a clear consensus about the follow-up of these small lesions is still missing [4,5,21], sometimes reaching critical positions [28].

## Conclusions

We might therefore conclude, returning to the points raised in the introduction, that a high preoperative detection rate of lesions presenting as an adrenal incidentaloma has been achieved in patients studied according to guidelines. Conversely, the unnecessary number of adrenalectomies performed in understudied patients, causing higher intra- and peri-operative morbidity, has not determined any significant change in the rate of primary adrenocortical cancer detected.

## Competing interests

Authors Mario Musella, Giovanni Conzo, Marco Milone, Francesco Corcione, Giulio Belli, Maurizio De Palma, Annunziato Tricarico, Luigi Santini, Antonietta Palazzo, Paolo Bianco, Bernadette Biondi, Rosario Pivonello, and Annamaria Colao:

1. In the past five years have not received reimbursements, fees, funding, or salary from an organization that may in any way gain or lose financially from the publication of this manuscript, either now or in the future.

2. Do not hold any stocks or shares in an organization that may in any way gain or lose financially from the publication of this manuscript, either now or in the future.

3. Do not hold or are not currently applying for any patents relating to the content of the manuscript.

4. Have not received reimbursements, fees, funding, or salary from an organization that holds or has applied for patents relating to the content of the manuscript.

5. Do not have any other financial competing interests or financial ties to disclose.

6. Do not have non-financial competing interests (political, personal, religious, ideological, academic, intellectual, commercial or any other) to declare in relation to this manuscript.

## Authors' contributions

MM, GC, FC, GB, MDP, AT, and LS, have made substantial contributions to conception and design of the study and acquisition of data by performing all the adrenalectomies reported. MM, MM, PB, and RP made the acquisition of data by filling and checking the form of each patient and performed the statistical analysis. BB, RP and AC, analyzed and interpreted data by checking the endocrine laboratory assays and the CT scan or MRI reports. MM, MM, GC and RP, have been involved in drafting the manuscript and revising it critically. All Authors have given final approval of the version to be published.

## Pre-publication history

The pre-publication history for this paper can be accessed here:

http://www.biomedcentral.com/1471-2482/13/57/prepub

## References

[B1] Bittner JB Brunt ML Evaluation and management of adrenal incidentaloma J Surg Oncol 2012 106 557 564 10.1002/jso.23161 22623268

[B2] Kanthan R Senger JL Kanthan S Three uncommon adrenal incidentalomas: a 13-year surgical pathology review World J Surg Oncol 2012 10 64 10.1186/1477-7819-10-64 22540324 PMC3407001

[B3] Mazzaglia PJ Monchik JM Limited value of adrenal biopsy in the evaluation of adrenal neoplasm: a decade of experience Arch Surg 2009 144 465 470 10.1001/archsurg.2009.59 19451490

[B4] NIH State-of-the-Science Statement on management of the clinically inapparent adrenal mass (“incidentaloma”) NIH Consens State Sci Statements 2002 19 2 1 25 14768652

[B5] Zeiger MA Thompson GB Duh QY Hamrahian AH Angelos P Elaraj D The American Association of Clinical Endocrinologists and American Association of Endocrine Surgeons medical guidelines for the management of adrenal incidentalomas Endocr Pract 2009 15 Suppl 1 1 20 19632967 10.4158/EP.15.S1.1

[B6] Smith CD Weber CJ Amerson JR Laparoscopic adrenalectomy: new gold standard World J Surg 1999 23 389 396 10.1007/PL00012314 10030863

[B7] Mantero F Terzolo M Arnaldi G A survey on adrenal incidentaloma in Italy. Study group on adrenal tumours of the Italian society of endocrinology J Clin Endocrinol Metab 2000 85 637 644 10.1210/jc.85.2.637 10690869

[B8] Grumbach MM Biller BM Braunstein GD Management of the clinically inapparent adrenal mass (“incidentaloma”) Ann Intern Med 2003 138 424 429 10.7326/0003-4819-138-5-200303040-00013 12614096

[B9] Shen WT Sturgeon C Duh QY From incidentaloma to Adrenocortical carcinoma: the surgical management of adrenal tumors J Surg Oncol 2005 89 186 192 10.1002/jso.20180 15719374

[B10] Mitchell IC Nwariaku FE Adrenal masses in the cancer patient: surveillance or excision Oncologist 2007 12 168 174 10.1634/theoncologist.12-2-168 17296812

[B11] Miccoli P Raffaelli M Berti P Adrenal surgery before and after the introduction of laparoscopic adrenalectomy Br J Surg 2002 89 779 782 10.1046/j.1365-2168.2002.02110.x 12027991

[B12] Saunders BD Wainess RM Dimick JB Trends in utilization of adrenalectomy in the United States: have indications changed? World J Surg 2004 28 1169 1175 10.1007/s00268-004-7619-6 15490057

[B13] Conzo G Tricarico A Belli G Adrenal incidentalomas in the laparoscopic era: the role of a correct surgical indication. Observations from 255 consecutive adrenalectomies in an Italian series Can J Surg 2009 52 6 E281 E285 20011165 PMC2792399

[B14] Boland GWL Blake MA Hahn PF Mayo-Smith WW Incidental adrenal lesions: principles, techniques, and algorithms for imaging characterization Radiology 2008 249 756 775 10.1148/radiol.2493070976 19011181

[B15] Hamrahian AH Ioachimescu AG Remer EM Clinical utility of noncontrast computed tomography atten- uation value (Hounsfield units) to differentiate adrenal ade- nomas/hyperplasias from nonadenomas: cleveland clinic experience J Clin Endocrinol Metab 2005 90 871 877 15572420 10.1210/jc.2004-1627

[B16] Terzolo M Stigliano A Chiodini I Loli P Furlani L Arnaldi G Reimondo G Pia A Toscano V Zini M Borretta G Papini E Garofalo P Allolio B Dupas B Mantero F Tabarin A AME position statement on adrenal incidentaloma Eur J Endocrinol 2011 164 851 870 10.1530/EJE-10-1147 21471169

[B17] De Leo M Cozzolino A Colao A Pivonello R Subclinical Cushing’s syndrome Best Pract Res Clin Endocrinol Metab 2012 26 497 505 10.1016/j.beem.2012.02.001 22863391

[B18] Conzo G Musella M Corcione F De Palma M Ferraro F Palazzo A Napolitano S Milone M Pasquali D Sinisi AA Colantuoni V Santini L Laparoscopic adrenalectomy, a safe procedure for pheochromocytoma. A retrospective review of clinical series Int J Surg 2013 11 152 156 10.1016/j.ijsu.2012.12.007 23267853

[B19] Grogan RH Mitmaker E Vriens MR Adrenal incidentaloma: does an adequate workup rule out surprises? Surgery 2010 148 392 397 10.1016/j.surg.2010.05.001 20576282

[B20] Lumachi F Basso SM Borsato S Tregnaghi A Zucchetta P Marzola MC Cecchin D Bui F Favia G Role and cost-effectiveness of adrenal imaging and image guided FNA cytology in the management of incidentally discovered adrenal tumours Anticancer Res 2005 25 4559 4562 16334141

[B21] Mazzaglia PJ Vezeridis MP Laparoscopic adrenalectomy: balancing the operative indications with the technical advances J Surg Oncol 2010 101 739 744 10.1002/jso.21565 20512951

[B22] Sturgeon C Shen WT Clark OH Duh QY Kebebew E Risk assesment in 457 adrenal cortical carcinomas: how much does tumor size predict the likelihood of malignancy? J Am Coll Surg 2006 202 423 430 10.1016/j.jamcollsurg.2005.11.005 16500246

[B23] Lau H Lo CY Lam KY Surgical implications of underestimation of adrenal tumor size by computed tomography Br J Surg 1999 186 385 387 10201784 10.1046/j.1365-2168.1999.01048.x

[B24] Assalia A Gagner M Laparoscopic adrenalectomy Br J Surg 2004 91 1259 1274 10.1002/bjs.4738 15376201

[B25] O’Neill CJ Spence A Logan B Suliburk JW Soon PS Learoyd DL Sidhu SB Sywak MS Adrenal Incidentalomas: risk of adrenocortical carcinoma and clinical outcomes J Surg Oncol 2010 102 450 453 10.1002/jso.21553 20734420

[B26] Kasperlik-Zaluska AA Otto M Cichocki A Incidentally discovered adrenal tumors: a lesson from observation of 1444 patients Horm Metab Res 2008 40 338 341 10.1055/s-2008-1073167 18491253

[B27] Wang ST Cheung K Sanziana AR Sosa JA A cost-effectiveness analysis of adrenalectomy for nonfunctional adrenal incidentalomas: Is there a size threshold for resection? Surgery 2012 152 1125 1132 10.1016/j.surg.2012.08.011 22989893

[B28] Cawood TJ Hunt PJ O’Shea D Cole D Soule S Recommended evaluation of adrenal incidentalomas is costly, has high false positive rates and confers a risk of fatal cancer that is similar to the risk of the adrenal lesion becoming malignant, time for a rethink? Eur J Endocrinol 2009 161 513 527 10.1530/EJE-09-0234 19439510

